# Enhancing COVID-19 Risk Mitigation in Bisha City Primary Schools: Investigating Teacher and Administrative Staff Awareness Through a Comprehensive Survey

**DOI:** 10.7759/cureus.61337

**Published:** 2024-05-29

**Authors:** Ibrahim A Eljack, Hanan Sakr, Abdullah Alhalafi, Abdullah A Alsoloule, Alyazeed A Alsamhoud, Moath A Suhaim, Mohammed T Alshehri, Salah A Aljuhani, Bashar M Alelyani, Malik M Alamri

**Affiliations:** 1 Department of Family and Community Medicine, College of Medicine, University of Bisha, Bisha, SAU; 2 Department of Child Health, College of Medicine, University of Bisha, Bisha, SAU; 3 College of Medicine, University of Bisha, Bisha, SAU

**Keywords:** administrative, bisha city, covid-19, investigating teacher, primary schools, risk mitigation, staff awareness

## Abstract

Background and aims

This research investigates COVID-19 awareness among primary school teachers and staff in Bisha City. It aims to enhance safety protocols by examining knowledge, awareness levels, and demographic associations. Despite school reopening, concerns linger. The study promotes informed decision-making, fostering a safer school environment and contributing to the well-being of the educational community.

Methods

In an institutional-based cross-sectional study among primary school teachers and administrative staff in Bisha City, our research aimed to comprehensively evaluate awareness regarding specific measures for minimizing COVID-19 risks. With a sample size of 348 participants, we employed a robust methodology, including online questionnaires addressing sociodemographic characteristics and knowledge about COVID-19 risks. The data collection period spanned from March 2022 to December 2023, providing a temporal context for responses. A pilot test ensured questionnaire clarity, and efforts were made to enhance reliability and validity, incorporating validated scales and iterative adjustments based on feedback. Non-response or incomplete responses were handled transparently, with sensitivity analyses to assess potential impact. The awareness level was measured using 17 Likert scale questions, and predefined categories (poor, moderate, and good) facilitated result interpretation. Researcher influence was minimized through training and inter-rater reliability checks. Confidentiality and anonymity were rigorously maintained, adhering to ethical considerations. Statistical analyses employed frequency tables, percentages, mean, standard deviations, and the chi-square test. Dissemination included academic publications, reports to the educational directorate, and presentations at conferences. This holistic approach contributes to the robustness and societal impact of our study, offering insights into COVID-19 awareness among educators in Bisha City.

Results

In this study assessing awareness among teachers and administrative staff in Bisha City regarding COVID-19 risk minimization, data from 348 respondents revealed key bio-demographic characteristics. The majority demonstrated good knowledge of environmental (83%) and personal hygiene risks (84%). The chi-square test indicated no significant associations between bio-demographic factors and awareness levels. Specifically, for age groups, χ²(4, N = 348) = 5.46, p = 0.707; for gender, χ²(1, N = 348) = 1.95, p = 0.744; for educational levels, χ²(4, N = 348) = 2.13, p = 0.995; for residency, χ²(1, N = 348) = 1.11, p = 0.892; and for job types, χ²(3, N = 348) = 8.30, p = 0.404. The absence of significant associations underscores the potential universality of successful awareness campaigns, suggesting that future efforts can maintain an inclusive approach without tailoring messages. These results emphasize the importance of sustained awareness efforts across the diverse demographic spectrum of the educational community.

Conclusion

This study reveals robust COVID-19 awareness among primary school teachers and staff in Bisha City, with no significant demographic associations. Successful, inclusive awareness campaigns can further enhance safety measures and promote well-being in the educational community.

## Introduction

The clinical manifestations of COVID-19 in children, often benign, raise concerns due to potential asymptomatic but infectious cases [1]. Globally, countries implemented community containment, notably school closures, despite inconclusive evidence of their efficacy [2]. Derived from past influenza outbreaks where children facilitated viral transmission, these measures, influenced by available data, were universally implemented [3].

School closures pose adverse effects on individuals and communities, impacting learning, especially in primary schools essential for fundamental skills [4]. Additionally, reduced physical activity and potential negative effects on mental health necessitate reopening schools. Despite concerns from families and the community about infection risks, primary schools in Saudi Arabia have reopened after a two-year closure. However, worries about the outbreak persist among families, students, and teachers [5].

For instance, recent surveys indicate that the majority of families express ongoing concerns about the safety of sending their children back to school, highlighting the need for transparent communication and evidence-based strategies to address these worries [6].

Our research endeavors to comprehensively evaluate the awareness among teachers and administrative staff in primary schools in Bisha City concerning specific measures geared toward minimizing the risks associated with COVID-19 infection. Our overarching goal is to not only assess this awareness but also to emphasize its societal impact.

In pursuit of this general objective, we have delineated specific objectives that guide our study. First, we aim to delve into the knowledge possessed by teachers and administrative staff regarding the specific risk factors intricately linked with COVID-19 infection in primary schools. This understanding is anticipated to contribute significantly to informed decision-making processes, ultimately promoting the overall well-being of the community. Secondly, our focus extends to assessing the awareness levels of teachers and administrative staff regarding the precise measures that are strategically designed to minimize the risks of COVID-19 infection within primary school settings. By doing so, we intend to foster a school environment that is not only safer but also conducive to the holistic health of both students and staff. Finally, our research seeks to explore the association between the varying levels of awareness exhibited by teachers and administrative staff and their respective sociodemographic data. This exploration is poised to facilitate targeted awareness campaigns and interventions, tailored to specific demographic groups, thereby ensuring a more effective dissemination of crucial information. Through these objectives, our research aspires to contribute meaningfully to the well-being and safety of the educational community in Bisha City.

## Materials and methods

Methodology

An institutional-based cross-sectional study was conducted among teachers and administrative staff in primary schools in Bisha City. Bisha, located in the southwestern Saudi Arabian province of 'Asir, had a population of 205,346 according to the 2010 Census. The city comprises 91 primary schools, with 37 schools designated for males and 54 for females. The total number of teachers and administrative staff in these schools is 1936, consisting of 950 males and 986 females. The study included teachers and administrative staff in both public and private primary schools in Bisha City.

Inclusion Criteria

Teachers working in primary schools in Bisha. Administrative staff working in primary schools in Bisha.

Exclusion Criteria

Students. Teachers or administrative staff working outside Bisha City. Teachers and administrative staff in intermediate and secondary schools. Teachers or administrative staff who were not accessible during data collection.

Sample Size and Sampling Technique

The sample size was determined using the Roasoft sample size calculator, yielding 321 participants. To enhance the study's power, the sample size was increased to 350. Simple random sampling was employed to select primary schools in Bisha City. Subsequently, all teachers and administrative staff in each school were included to complete the questionnaire.

Data collection

An online questionnaire, designed using Google Forms, was utilized to collect data. The questionnaire addressed sociodemographic characteristics, knowledge about the risks of COVID-19 infection, and the awareness of teachers and administrative staff regarding measures to minimize the chances of COVID-19 infection. The data collection period for this study spanned 3/2022 to 12/2023. This temporal framework is crucial in providing readers with a clear understanding of the study's timeline, allowing insights into any potential variations in responses that may be influenced by external factors during that period.

Pilot testing

Before embarking on the main data collection, a meticulous pilot test of the questionnaire was conducted to fine-tune its clarity and comprehensibility. A small group of individuals, distinct from our study participants, actively participated in this preliminary phase. The primary objective was to identify and rectify any potential issues related to the wording of questions or the understanding of respondents. The invaluable feedback received from the pilot test participants facilitated crucial refinements and adjustments to ensure the questionnaire's efficacy. This iterative process not only enhances the reliability of our data collection instrument but also reinforces the validity of our study by proactively addressing any ambiguities that might arise during the main data collection phase.

Reliability and validity of the questionnaire

In our commitment to robust and meaningful research, we took deliberate steps to ensure the reliability and validity of our questionnaire. Before the main data collection, the questionnaire underwent a thorough pre-testing phase. A select group of individuals, distinct from our study participants, actively participated in this preliminary evaluation. Their valuable feedback enabled us to identify and address any potential issues related to question clarity and respondent understanding. Adjustments were meticulously made based on this feedback, refining the questionnaire for optimal effectiveness. Additionally, to bolster the questionnaire's validity, we incorporated validated scales where applicable. This comprehensive approach not only enhances the overall quality of our data collection but also underscores our dedication to producing insightful and trustworthy research outcomes.

Handling non-response or incomplete responses

In the event of non-response or incomplete responses, a meticulous approach is adopted to minimize potential biases and uphold the study's validity. Efforts were made to encourage full participation during data collection, and respondents were informed about the importance of providing comprehensive and accurate information. If non-response or incomplete responses occur, the researchers analyze the available data and assess any patterns or trends. A detailed record of non-responses and incomplete responses was maintained, and sensitivity analyses were conducted to evaluate the potential impact on the study's outcomes. This transparent approach ensured a thorough understanding of the data's limitations and contributed to informed interpretations.

Measurement of level of awareness

Seventeen Likert scale questions with five answer options (ranging from highly disagree to highly agree) were utilized to measure the level of awareness. Results were categorized into three levels: poor awareness (below 60%), moderate awareness (60% to 74%), and good awareness (75% and above). The levels of awareness in this study, categorized as poor, moderate, and good, were predefined to facilitate a clear interpretation of the results. The rationale for selecting these categories is rooted in establishing meaningful distinctions in the participants' understanding of COVID-19 risks and preventive measures.

Rationale

Poor Awareness (Below 60%)

Participants falling into this category exhibit a lower level of awareness, indicating a significant gap in understanding COVID-19 risks and mitigation strategies. This threshold ensures a distinct classification of respondents with a clear need for targeted interventions and educational initiatives.

Moderate Awareness (60% to 74%)

This category represents a mid-range level of awareness, indicating a moderate understanding of COVID-19-related factors. Respondents in this group may benefit from additional educational efforts to enhance their awareness and reinforce key preventive measures.

Good Awareness (75% and Above)

Participants in this category demonstrate a high level of awareness, signifying a robust understanding of COVID-19 risks and effective preventive measures. This classification identifies individuals who can serve as potential advocates for promoting awareness within their respective communities. These predefined levels are designed to offer a practical and actionable framework for interpreting the survey results. They enable a nuanced assessment of the participants' awareness, guiding the development of targeted interventions based on the specific needs identified within each awareness category. This approach ensures a meaningful analysis of the data and contributes to the effectiveness of future awareness campaigns.

Mitigating researcher influence

To ensure objectivity and minimize potential biases in the study, specific measures were implemented throughout the research process. The research team underwent comprehensive training to maintain a consistent approach to data collection and analysis. During the questionnaire administration, emphasis was placed on neutrality, and participants were encouraged to express their views candidly. Additionally, inter-rater reliability checks were conducted regularly among the research team members involved in data analysis. This approach aimed to identify and address any divergences in interpretation, contributing to the overall reliability of the study findings. Transparency in reporting methodologies and findings further reinforced the study's credibility and minimized the impact of potential researcher influence.

Researchers training

Before the commencement of data collection, all researchers involved underwent a comprehensive training program to ensure consistency and standardization in the approach. The training encompassed an in-depth understanding of the research objectives, familiarity with the questionnaire, and guidance on interacting with study participants. Emphasis was placed on maintaining a neutral and unbiased demeanor to minimize potential interviewer effects. Practical sessions, including mock interviews and scenario-based exercises, were conducted to enhance the researchers' skills in handling various situations during data collection. This training initiative aimed to uphold the integrity of the study by promoting a uniform and rigorous data collection process across the entire research team.

Ensuring confidentiality and anonymity

Confidentiality and anonymity of the participants were rigorously safeguarded throughout the study. All collected data, particularly sensitive information related to participants' knowledge and awareness regarding COVID-19, were stored securely and accessible only to the research team. Personal identifiers were replaced with unique codes, ensuring that individual responses could not be traced back to specific participants. The research team adhered to strict ethical standards, and any information disclosed by participants was used exclusively for research purposes. Moreover, all team members involved in data analysis and interpretation were sensitized to the importance of maintaining confidentiality and fostering a secure environment for participants' contributions.

Ethical considerations

A written letter was dispatched to the educational directorate, and a consent letter was obtained from each respondent (consent letter attached in Appendix 1). The study adhered to ethical standards and protocols to ensure the privacy and well-being of the participants.

Statistical analysis

Frequency tables and percentages were employed to present descriptive variables. Mean and standard deviations were calculated for numerical variables, offering insights into central tendency and variability. Applied to analyze categorical variables, the chi-square test determined the associations between dependent and independent variables. Significance was established when p-values were less than 0.05.

Dissemination of results

The findings of this study were disseminated through various channels to ensure broader accessibility and impact. Academic publications in peer-reviewed journals served as a primary means to share the results with the scholarly community. Additionally, comprehensive reports summarizing the key outcomes were submitted to the educational directorate, providing valuable insights for educational policymakers. Furthermore, the research team presented the study outcomes at relevant conferences, fostering discussions and sharing knowledge within the academic and educational spheres. This multipronged approach aimed to maximize the reach and influence of the study, contributing to both academic discourse and practical applications in the field.

## Results

Data collected from 348 respondents, achieving a response rate of 99.4%, revealed key bio-demographic characteristics as shown in Table 1. The mean age was 43.28 with a standard deviation of 7.82; 83.4% fell within the age group of 35-55. Males comprised 71% of the participants, while females comprised 29%. Teachers represented 81.7% of the sample, and 79.4% held a bachelor's degree. The majority resided in urban areas (81.7%).

**Table 1:** Bio-demographic data of the participants in this study.

Variable	Frequency (N=348)	Percentage
Age group:		
Less than 25	9	2.6
25-34	27	7.7
35-44	142	40.7
45-55	147	42.7
More than 55	21	6
Gender:		
Male	248	71
Female	100	29
Education level:		
High school	16	4.6
Diploma	35	10
Bachelor's	277	79.4
Master's	16	4.6
PhD	4	1.1
Residency:		
Rural	85	24.4
Urban	263	75.4
Type of the job:		
Administrative staff	31	8.9
Teacher	285	81.7
Employee	8	2.3
Other	24	6.9

**Figure 1:**
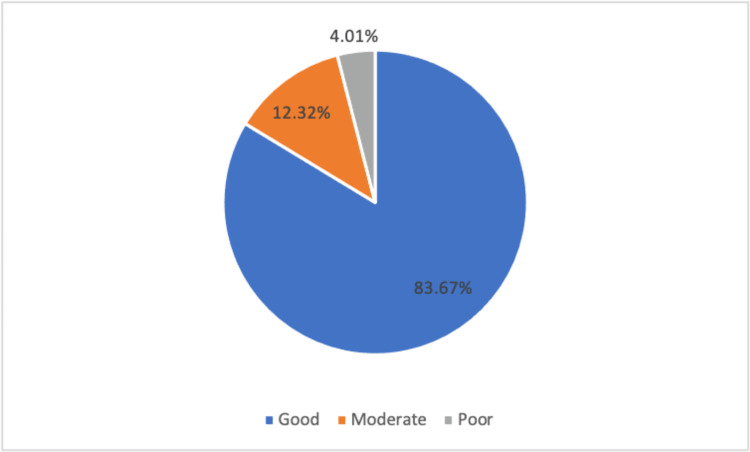
Level of knowledge about environmental risks of the participants in this study.

**Figure 2:**
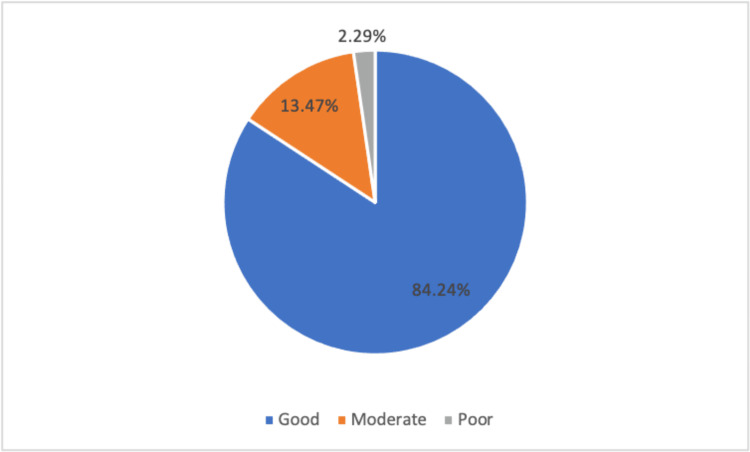
Level of knowledge about personal and personal hygiene risks of the participants in this study.

The assessment of knowledge about COVID-19 infection risks focused on environmental and personal hygiene factors. For environmental risks, questions probed the understanding of physical distance, crowding in closed areas, and the role of ventilation in risk reduction. Figure 1 indicated that 83% of participants demonstrated good knowledge of environmental risk factors. Figure 2 encapsulated personal and personal hygiene risks, encompassing questions on sharing objects, wearing masks, and vaccination efficiency. Results showed that 84% had good knowledge of personal hygiene risk factors.

**Figure 3:**
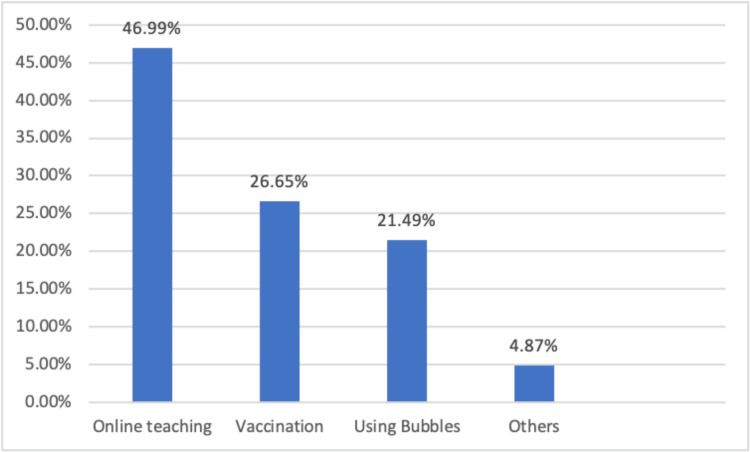
Respondents’ opinion of the best measures to minimize the risks of COVID-19 infection. Most of them (46%) prefer to reclose the schools and resume online teaching.

According to Figure 3, a majority of the respondents (46%) believe that the best way to minimize the risks of COVID-19 infection in primary schools is to reclose the schools and resume online teaching. Among the respondents, 26% and 21% suggest that vaccination and utilizing bubbles, respectively, would be the best ways to minimize the risk of COVID-19 infection in primary schools.

Table 2 presents the outcomes of the chi-square test examining the potential associations between various bio-demographic factors and the level of awareness among teachers and administrative staff concerning the mitigation of COVID-19 risks in primary school settings in Bisha City during 2022-2023. The chi-square value of 5.46 with a corresponding p-value of 0.707 indicates that there is no statistically significant association between different age groups and the level of awareness among participants. In other words, the age distribution of the respondents does not appear to influence their awareness of measures to minimize COVID-19 risks. With a chi-square value of 1.95 and a p-value of 0.744, the results suggest that gender does not significantly impact the level of awareness. Both male and female participants exhibit similar levels of awareness regarding COVID-19 risk mitigation measures.

**Table 2:** Association between bio-demographic factors and the level of awareness about how to minimize the risks of COVID-19 infection of the participants in this study.

Variable	Chi-square test value	p-Value
Age group	5.46	0.707
Gender	1.95	0.744
Educational level	2.13	0.995
Residency	1.11	0.892
Type of job	8.30	0.404

The chi-square value of 2.13 and a p-value of 0.995 indicate that there is no significant association between the educational level of participants and their awareness levels. Individuals with varying educational backgrounds seem to demonstrate similar levels of awareness regarding COVID-19 risks. The chi-square value of 1.11 and a p-value of 0.892 suggest that there is no statistically significant association between the participants' residency (urban or rural) and their level of awareness. Both urban and rural residents show comparable awareness levels regarding COVID-19 risk minimization. The chi-square value of 8.30 and a p-value of 0.404 imply that there is no significant association between the type of job (administrative staff, teacher, employee, or other) and the level of awareness. Participants from different job categories exhibit similar levels of awareness concerning measures to minimize COVID-19 risks in primary school settings.

Chi-square test results indicate that, within the studied population, age group, gender, educational level, residency, and type of job do not significantly influence the level of awareness regarding the minimization of COVID-19 risks among teachers and administrative staff in primary schools in Bisha City during the specified period.

**Figure 4:**
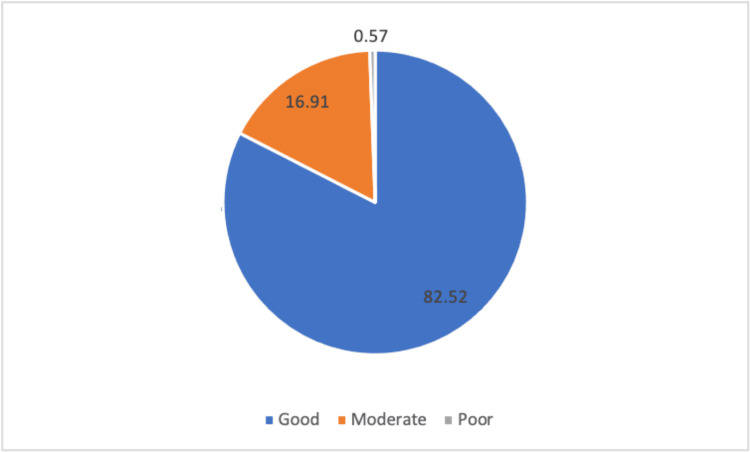
The level of awareness about minimizing the risks of COVID-19 infection of the participants in this study.

Figure 4 depicts the participants' level of awareness about minimizing COVID-19 infection risks, with 82% exhibiting good awareness. An analysis of the association between bio-demographic factors and awareness demonstrated no significant correlations, as indicated in Table 2.

The absence of significant associations between bio-demographic factors and awareness levels among teachers and administrative staff regarding the minimization of COVID-19 risks in primary schools in Bisha City carries important implications. First, it suggests that regardless of age, gender, educational level, residency, or type of job, participants exhibited a consistent level of awareness about COVID-19 risk mitigation measures. This uniformity is noteworthy as it indicates that awareness campaigns and educational interventions implemented in the region have likely been effective across diverse demographic groups.

Moreover, the lack of association underscores the potential universality of the awareness programs, indicating that these initiatives were successful in reaching and resonating with a broad spectrum of individuals within the educational community. This finding is encouraging from a public health perspective, as it implies that educational efforts have been inclusive, leaving no particular demographic subgroup less informed about essential COVID-19 risk mitigation measures.

In practical terms, these results suggest that future awareness campaigns and interventions can continue to adopt a broad approach without the need for tailoring messages based on specific bio-demographic characteristics. However, ongoing efforts to sustain and enhance awareness should remain a priority to ensure that the level of knowledge and adherence to preventive measures remain high across all segments of the educational workforce.

## Discussion

This study aimed to assess the awareness of teachers and administrative staff about minimizing the risks of COVID-19 infection. Based on the data collected, it can be concluded that the teachers and administrative staff had good knowledge regarding personal and environmental risks. They also showed a good level of awareness about how to minimize the risk of COVID-19 infection. The results revealed no significant association between biodemographic data and the level of awareness about minimizing the risks of COVID-19 infection. A high response rate (99%) was obvious in our study, and this finding undoubtedly reflects the high level of awareness of academic and administrative staff at primary schools toward their social responsibility and their role in supporting scientific research [7]. A study in Saudi Arabia done by Al-hanawi showed that the level of knowledge among the public participants was 81.64% [8]. The level of knowledge assessed in our study showed a similar result. The significantly high level of awareness of participants about different aspects related to COVID-19 infection evaluated in our research can simply be explained by the fact that COVID-19 infection and its preventive strategies were the hottest topics and pivotal issues in all social communication and broadcasting media [9]. Every single person was searching daily for updated modalities in the management and prevention of the pandemic to protect himself and his beloved family. No significant association between awareness level and different sociodemographic factors augments our theory that knowledge about the pandemic is not gender or academic-level dependent as information was globally distributed to every person through social and broadcast media [10]. We thought that this information gave us the green light that taking advantage of school openings is warranted. It was surprising that most of our participants chose to reclose schools and return to online conduction of academic classes as the best strategy to prevent the disease despite their high awareness status [11]. This probably can be explained first by their concerns and phobia of the lack of implementation of preventive strategies in primary school settings. The second issue that may drive them toward preferring online teaching is the abnormally high increase in case numbers during the current era; however, the emergence of the omicron strain with its highly contagious property was probably the main cause of the increased case number [12].

The unexpected finding that a majority of participants favored the reclosure of schools despite their high awareness levels of minimizing COVID-19 risks demands a thorough analysis. Several external factors may underlie this preference. The prevailing sentiments and perceptions within the local community, influenced by media coverage and government guidelines, could play a significant role [13]. If there are uncertainties or changes in official directives regarding school operations during the pandemic, individuals might favor a cautious approach aligned with these guidelines. Additionally, experiences with previous waves of the pandemic and the associated disruptions within the school community may shape participants' preferences, leading them toward conservative measures such as school reclosure. Overall, this unexpected trend highlights the intricate interplay of community, government, and individual factors in shaping preferences amidst the ongoing challenges posed by the COVID-19 pandemic [14].

While the overall knowledge levels among participants regarding minimizing COVID-19 risks are reported as high, it is essential to explore potential variability within distinct participant groups. Examining knowledge disparities among subgroups, such as teachers versus administrative staff or different age groups, can provide valuable insights. Variability may arise due to the nature of roles, responsibilities, and personal experiences within these subsets [15]. A more nuanced analysis can uncover specific areas of strength or potential gaps in awareness, contributing to a comprehensive understanding of the diverse knowledge landscape among participants. This exploration is crucial for tailoring targeted interventions and ensuring a more equitable dissemination of crucial information across all participant categories [16].

Acknowledging the substantial influence of media on participants' awareness levels regarding COVID-19 risks, it is crucial to scrutinize how media coverage might have shaped perceptions. While media plays a pivotal role in disseminating information, it is not without potential drawbacks and biases [17]. The intensity and framing of coverage can impact public perceptions, potentially amplifying certain aspects or creating undue fears. Biases in reporting, including sensationalism or selective emphasis, may contribute to an uneven understanding of the pandemic. Moreover, media channels may prioritize attention-grabbing content over nuanced, accurate information, potentially skewing participants' perspectives. A critical examination of the potential drawbacks and biases in the information disseminated through media channels is vital for interpreting participants' high awareness levels and ensuring a more informed and balanced public response to the ongoing challenges posed by COVID-19 [18].

The absence of a significant association between sociodemographic factors and awareness levels among participants regarding COVID-19 risks has significant implications. This finding suggests that knowledge about minimizing the risks of COVID-19 infection is universally high, irrespective of gender or academic level. The high awareness levels across diverse sociodemographic groups underscore the effectiveness of widespread information dissemination strategies, likely facilitated by global media coverage and public health campaigns [19].

This universal awareness implies that targeted educational strategies in school settings should be comprehensive and inclusive, addressing the entire academic community without prioritizing specific demographic groups. The uniformity in awareness levels suggests that information about the pandemic has been broadly accessible and well-distributed, transcending gender or academic distinctions. Educational initiatives should, therefore, focus on reinforcing existing knowledge, ensuring consistent understanding, and fostering a collective commitment to preventive measures [20].

Furthermore, the lack of sociodemographic disparities in awareness challenges assumptions that certain groups may be less informed. This calls for a nuanced approach to designing educational interventions, moving beyond stereotypical assumptions and tailoring strategies to address the unique needs of diverse individuals within the academic community [21].

This study is subject to several limitations that warrant consideration. First, the cross-sectional design impedes the establishment of causal relationships, limiting our ability to discern temporal patterns in knowledge acquisition. The reliance on self-reported data introduces the potential for response bias, and the exclusive focus on a single city restricts the generalizability of findings to broader geographical contexts. Additionally, the analysis employed a specific set of sociodemographic variables, overlooking potential influences such as socioeconomic status or cultural background. These limitations underscore the need for caution in interpreting the findings and call for future research efforts to address these constraints.

To enhance the robustness of research in this domain, future studies should embrace a longitudinal approach to unravel temporal shifts in awareness levels and gauge the sustained impact of educational interventions. The integration of mixed-methods approaches, combining qualitative insights with quantitative data, can offer a more comprehensive understanding of participants' knowledge and decision-making processes. Furthermore, there is a need for intervention studies to evaluate the effectiveness of targeted educational programs in influencing knowledge and preventive behaviors. Comparative analyses across diverse geographical regions and an exploration of information sources can deepen our understanding of global and regional disparities in COVID-19 awareness. Finally, in-depth investigations into the factors shaping participants' preferences, particularly regarding school reclosure, will provide valuable insights for shaping public health strategies in educational settings and beyond.

## Conclusions

Inarguably, the imperative to reopen schools after a two-year hiatus is undeniable, given its profound effects on primary school learning and the associated physical and mental consequences. Yet, lingering concerns persist among families and communities regarding the safety of their children in the educational environment. Our study aimed to comprehensively assess the knowledge and awareness levels of teachers and administrative staff concerning the mitigation of COVID-19 infection risks. The positive outcomes, where a significant majority demonstrated commendable knowledge and awareness, suggest the efficacy of ongoing educational efforts in alleviating concerns about the return to school. However, the prevalent preference for online teaching among educators as a risk reduction measure demands strategic intervention. To substantiate our findings and gauge the genuine impact of knowledge and awareness on COVID-19 infection risks, we advocate for rigorous follow-up studies, including the enumeration of actual infection cases among primary school students. In conclusion, our research not only underscores the need for informed policy and practice but also highlights the ongoing necessity for heightened awareness campaigns, aiming to safeguard the educational environment and the broader community.

## Appendices

Consent letter

Research title: Assess the Awareness of Teachers and Administrative Staff about Minimizing Risks of COVID-19 Infection in Primary School Settings in Bisha City in 2022.

You have been invited to be part of a research study to assess the extent of knowledge of male and female teachers and administrative staff in primary schools in the city of Bisha about ways to limit the spread of COVID-19 infection in primary schools. Thus, you were chosen to be a participant because of your teaching job in primary schools in the city of Bisha.

We ask you to read this form and you can ask any questions before agreeing to participate. The search does not include any private personal data.

This study is conducted by fourth-year students at the Faculty of Medicine, University of Bisha with their supervisors.

The purpose of this study is to assess the extent of knowledge of male and female teachers and administrative staff in primary schools in the city of Bisha about ways to limit the spread of the Coronavirus among school employees.

If you agree to participate, we would like to ask you to fill out the questionnaire that will be given to you.

Agree            (…..)

Not agree     (…..)

The questionnaire

Sociodemographic characteristics:

1- Age in years:

2- Gender: Male - Female

3- Educational level: High school - Diploma - Bachelor - Master - PhD

4- Residency: Rural - Urban

5- Type of job:  Administrative staff - Teacher - Employee - Other

Which of the following do you think is a risk of COVID-19?

No physical distance inside the class:

1 - Strongly disagree     2 - Disagree      3 - Neutral         4 - Agree      5 - Strongly agree

Crowding out - class, schoolyard, in entry and exits, school transports:

1 - Strongly disagree     2 - Disagree      3 - Neutral         4 - Agree      5 - Strongly agree

Poor ventilation of classrooms:

1 - Strongly disagree     2 - Disagree      3 - Neutral         4 - Agree      5 - Strongly agree

Poor hand hygiene:  

1 - Strongly disagree     2 - Disagree      3 - Neutral         4 - Agree      5 - Strongly agree

Sharing stuff:

1 - Strongly disagree     2 - Disagree      3 - Neutral         4 - Agree      5 - Strongly agree

Not wearing a face mask:

1 - Strongly disagree     2 - Disagree      3 - Neutral         4 - Agree      5 - Strongly agree

Students not vaccinated:

1 - Strongly disagree     2 - Disagree      3 - Neutral         4 - Agree      5 - Strongly agree

What is the best modality to minimize the risks of COVID-19?

1 - Vaccination of all students 2 - Using bubbles and grouping 3 - Closure of schools/using online teaching

How to reduce the environmental risks:

Bubbles will reduce the risks

1 - Strongly disagree     2 - Disagree      3 - Neutral         4 - Agree      5 - Strongly agree

The application of physical distance in the classroom will minimize the risks

1 - Strongly disagree     2 - Disagree      3 - Neutral         4 - Agree      5 - Strongly agree

Good classroom ventilation (open windows) will minimize the risks

1 - Strongly disagree     2 - Disagree      3 - Neutral         4 - Agree      5 - Strongly agree

Holding out-of-class activities will minimize the risks

1 - Strongly disagree     2 - Disagree      3 - Neutral         4 - Agree      5 - Strongly agree

Regular daily cleaning and disinfection of the school environment will minimize the risks

1 - Strongly disagree     2 - Disagree      3 - Neutral         4 - Agree      5 - Strongly agree

How to minimize personal risks:

Providing tools for hand hygiene and soaps will minimize the risk

1 - Strongly disagree     2 - Disagree      3 - Neutral         4 - Agree      5 - Strongly agree

Wearing the face mask among the teachers will minimize the risk of COVID-19

1 - Strongly disagree     2 - Disagree      3 - Neutral         4 - Agree      5 - Strongly agree

Promoting respiratory etiquette will minimize the risk

1 - Strongly disagree     2 - Disagree      3 - Neutral         4 - Agree      5 - Strongly agree

Awareness about dealing with COVID-19 students:

Any student who had contact with the COVID-19 patient should be isolated

1 - Strongly disagree     2 - Disagree      3 - Neutral         4 - Agree      5 - Strongly agree

## References

[1] Alharbi M, Kazzaz YM, Hameed T (2021). SARS-CoV-2 infection in children, clinical characteristics, diagnostic findings and therapeutic interventions at a tertiary care center in Riyadh, Saudi Arabia. J Infect Public Health.

[2] Shankar PR, Palaian S, Vannal V, Sreeramareddy CT (2021). Non-pharmacological infection prevention and control interventions in COVID-19: What does the current evidence say?. Int J Prev Med.

[3] Saunders-Hastings PR, Krewski D (2016). Reviewing the history of pandemic influenza: Understanding patterns of emergence and transmission. Pathogens.

[4] James M, Marchant E, Defeyter MA, Woodside J, Brophy S (2021). Impact of school closures on the health and well-being of primary school children in Wales UK: A routine data linkage study using the HAPPEN Survey (2018-2020). BMJ Open.

[5] Mahfouz MS, Alqassim AY, Sobaikhi NH (2023). Physical activity, mental health, and quality of life among school students in the Jazan region of Saudi Arabia: A cross-sectional survey when returning to school after the COVID-19 pandemic. Healthcare (Basel).

[6] Bell J, Condren M (2016). Communication strategies for empowering and protecting children. J Pediatr Pharmacol Ther.

[7] Amro FM, Rayan AH, Eshah NF, ALashtawy MS (2022). Knowledge, attitude, and practices concerning Covid-19 preventive measures among healthcare providers in Jordan. SAGE Open Nurs.

[8] Al-Hanawi MK, Angawi K, Alshareef N (2020). Knowledge, attitude and practice toward COVID-19 among the public in the Kingdom of Saudi Arabia: A cross-sectional study. Front Public Health.

[9] Alanezi F, Aljahdali A, Alyousef S, Alrashed H, Alshaikh W, Mushcab H, Alanzi T (2020). Implications of public understanding of COVID-19 in Saudi Arabia for fostering effective communication through awareness framework. Front Public Health.

[10] Cipolletta S, Andreghetti GR, Mioni G (2022). Risk perception towards COVID-19: A systematic review and qualitative synthesis. Int J Environ Res Public Health.

[11] Neil-Sztramko SE, Caldwell H, Dobbins M (2021). School-based physical activity programs for promoting physical activity and fitness in children and adolescents aged 6 to 18. Cochrane Database Syst Rev.

[12] Lapitan LD Jr, Tiangco CE, Sumalinog DA, Sabarillo NS, Diaz JM (2021). An effective blended online teaching and learning strategy during the COVID-19 pandemic. Educ Chem Eng.

[13] Son C, Hegde S, Smith A, Wang X, Sasangohar F (2020). Effects of COVID-19 on college students' mental health in the United States: Interview survey study. J Med Internet Res.

[14] Martín-Sánchez M, Cáceres-Muñoz J, Flores-Rodríguez C (2022). The effects of the COVID-19 pandemic on educational communities: Evidence of early childhood education students. Int J Environ Res Public Health.

[15] Mantilla-Morrón M, Suárez-Palacio D, Urina-Triana MA (2023). The level of knowledge, attitudes, and practices in a Caribbean Colombian population that recovered from COVID-19 during the pandemic. Healthcare (Basel).

[16] Gopalkrishnan N (2018). Cultural diversity and mental health: Considerations for policy and practice. Front Public Health.

[17] Tune SN, Sarkar MM, Uddin MN, Pinto MZ, Ahmed SM (2021). Reactions to the media coverage during the first wave of COVID-19 in Bangladesh. Heliyon.

[18] Han R, Xu J, Pan D (2022). How media exposure, media trust, and media bias perception influence public evaluation of COVID-19 pandemic in international metropolises. Int J Environ Res Public Health.

[19] Al Abri ZG, Al Zeedi MA, Al Lawati AA (2021). Risk factors associated with COVID-19 infected healthcare workers in Muscat Governorate, Oman. J Prim Care Community Health.

[20] McGee BS, Germany AF, Phillips RL, Barros-Lane L (2021). Utilizing a critical race theory lens to reduce barriers to social and emotional learning: A call to action. Child Sch.

[21] Gaysynsky A, Heley K, Chou WS (2022). An overview of innovative approaches to support timely and agile health communication research and practice. Int J Environ Res Public Health.

